# Development of L1 Vertebral Anthropomorphic Model for Densitometric Phantom Improvement

**DOI:** 10.17691/stm2025.17.4.05

**Published:** 2025-08-29

**Authors:** A.V. Petraikin, A.M. Mikhailova, N.D. Kudryavtsev, M.V. Cherkasskaya, V.O. Yastrebova, O.V. Omelyanskaya, Y.A. Vasilev

**Affiliations:** MD, DSc, Associate Professor, Senior Researcher, Standardization and Quality Control Department; Scientific and Practical Center for Diagnostics and Telemedicine, Moscow City Healthcare Department, Bldg 1, 24 Petrovka St., Moscow, 127051, Russia; Junior Researcher, Innovative Technologies Department; Scientific and Practical Center for Diagnostics and Telemedicine, Moscow City Healthcare Department, Bldg 1, 24 Petrovka St., Moscow, 127051, Russia; Junior Researcher, Standardization and Quality Control Department; Scientific and Practical Center for Diagnostics and Telemedicine, Moscow City Healthcare Department, Bldg 1, 24 Petrovka St., Moscow, 127051, Russia; PhD, Researcher, Standardization and Quality Control Department; Scientific and Practical Center for Diagnostics and Telemedicine, Moscow City Healthcare Department, Bldg 1, 24 Petrovka St., Moscow, 127051, Russia; Junior Researcher, Standardization and Quality Control Department; Scientific and Practical Center for Diagnostics and Telemedicine, Moscow City Healthcare Department, Bldg 1, 24 Petrovka St., Moscow, 127051, Russia; Managing Director of Units of Science Directorate; Scientific and Practical Center for Diagnostics and Telemedicine, Moscow City Healthcare Department, Bldg 1, 24 Petrovka St., Moscow, 127051, Russia; PhD, Chief Medical Officer; Scientific and Practical Center for Diagnostics and Telemedicine, Moscow City Healthcare Department, Bldg 1, 24 Petrovka St., Moscow, 127051, Russia

**Keywords:** phantom, vertebra, densitometry, osteoporosis

## Abstract

**The aim of the study** was to develop L1 vertebral anthropomorphic model to improve the previously developed PHK FK2 phantom.

**Materials and Methods:**

The vertebra was made using 3D printing by a digital model obtained from DICOM files of the abdominal CT examination. The phantom construction consists of three layers of different X-ray density. In the base made of photopolymer resin there is a cylindrical recess filled with a plastic mixture to imitate the normal state of the spongy substance (high density), osteopenia (moderate density decrease) and osteoporosis (significant decrease in density). Mineral density is regulated by changing β-tricalcium phosphate concentration. The cortical layer is modelled by applying metal foil on the base surface.

**Results:**

In X-ray tube voltage of 120 kV, the mean square deviations of the measured X-ray density values of the vertebral body, spongy substance and the cortical layer were 12.40, 3.96, and 57.23 HU, respectively. The mineral density assessment of the spongy substance for three X-ray tube voltages (100, 120, 140 kV) showed the mean absolute error to be 7.4 mg/ml, and the mean relative error — 7.3% (variation coefficient). The correction coefficient equal 7 mg/ml was used to correct the values, and after using the coefficient the mean absolute error decreased up to 0.4 mg/ml, and the mean relative error — up to 0.4% (variation coefficient). The relative measurement errors of the ventral, medial and dorsal vertebral body dimensions were 3.6, 2.7, and 2.9%, respectively.

**Conclusion:**

The methods used in developing a vertebral model can be applied in modeling the entire range of the mineral density of bone spongy substance: from osteoporosis to norm. The developed model demonstrates high stability of X-ray characteristics and anatomical accuracy; therefore, it can be used for equipment calibration, quality control of diagnostic systems, and in the training process to demonstrate the bone structure changes.

## Introduction

In recent years, in diagnostic radiology and imaging studies, special attention is paid to equipment adjustment and calibration. Properly functioning equipment provides high reliability, accuracy, along with short- and high-term reproducibility of measurements [1]. Diagnostic equipment is adjusted using phantoms, which are specific objects imitating anatomical structures of the human body.

In the age of the rise of artificial intelligence (AI) algorithms to interpret medical images, phantoms can be effective tools for testing the accuracy of such algorithms [2]. For instance, the automatic analysis of CT images aimed to determine osteoporosis signs requires high reliability of the measurements of the mineral density of the bone tissue and vertebral body height [3, 4]. Phantoms enable to create controlled conditions, when it is possible to assess if AI algorithm can accurately take required measurements [5].

Previously, a group of authors from Scientific and Practical Center for Diagnostics and Telemedicine [6, 7] developed PHK FK2 phantom designed to assess the accuracy of densitometric studies in asynchronous quantitative computed tomography and dual energy X-ray absorptiometry. However, in standard PHK FK2 the vertebrae imitate simple geometric figures (cylinder and parallelepiped) that prevents from assessing the accuracy of AI algorithms.

**The aim of the present study** was to develop L1 vertebral anthropomorphic model to improve the previously developed PHK FK2 phantom.

## Materials and Methods

L1 was chosen as a modeling object, since the change in the bone mineral density (BMD) of the vertebra is the main marker of osteoporosis [8]. The developed model is a three-layer anthropomorphic demountable product consisting of the vertebral body, which is attached by means of two legs with an arch with two plates, and between them there are the processes made of photopolymer resin. The vertebral body has a cavity filled with modeling material with β-tricalcium phosphate (β-TCP) added, the latter simulating the spongy bone substance. The assembled model is coated with a copper foil layer to imitate the cortical layer of the vertebra.

### 3D-model building

**Figure 1.**
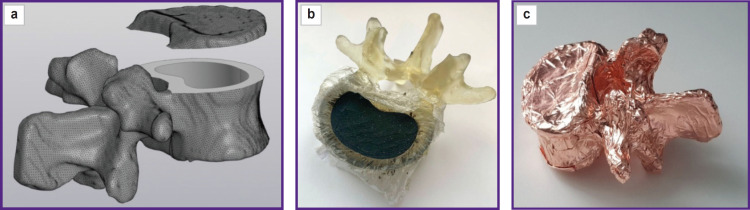
Anthropomorphic vertebral model consisting of the body, processes and the upper cortical plate (a); filled with plastic mixture (b); the resulting view, assembled model (c)

An anonymized abdominal CT examination was chosen as the reference. The data were imported into the software 3D Slicer, 5.2 version; we used a mask for marking the vertebrae, and saved the 3В-model in STL format. After that a STL-file was imported into the program Kompas-3D (Ascon, Russia) and formed a polygonal object consisting of 78,780 triangles. Then the object was processed for further modification, the resulting model was cut by the plane perpendicular to the longitudinal vertebral axis into two parts notionally named “vertebral body” and “plate” (Figure 1 (a)). The plate was from 3 to 6 mm thick. In the vertebral body we formed a cylindrical recess, which was filled with the spongy substance equivalent in such a way that the minimal wall thickness was 3 mm. To provide the connection and tightness, on the lower surface of the plate we made the projection 1.5 mm high. The program Meshmixer (Autodesk, USA) was used to check the obtained model and correct mistakes.

### Model basis making

The vertebral body and plate were made of photopolymer resin by means of LCD-printing using Elegoo Saturn 2 printer (Shenzhen Elegoo Technology Co., China). The time of parallel printing for both models was 5.5 h. Uncured resin residues were removed using ethyl alcohol followed by exposing the models to radiation in a UV furnace.

### Spongy substance imitation

The spongy substance of the vertebral body was imitated using the mixture of the composite modeling clay and β-TCP containing Ca_3_(PO_4_)_2_, its purity being over 98% (Sigma-Aldrich, USA). Such mixture is an equivalent of bone tissue hydroxyl apatite. The advantage of the technique is in possible change of the specified mineral density of the spongy substance by producing a set of replaceable insets with different β-TCP concentration.

As a base we chose the composite including the following components (most closely approximate to the natural composition of the bone spongy substance): starch (organic component), ceresin and paraffin (fatty component), and paraffin oil.

The target BMD value was chosen to be 100 mg/ml corresponding to osteopenia according to ACR 2023 criteria [9]. Using the program Kompas-3D we calculated the volume of the recess in the vertebral body (spongy substance volume) — 17.4 ml. The plastic composite density was experimentally measured — 1.25 g/ml, and the tapped density of β-TCP — 1.33 g/ml.

There were prepared 18 ml of the mixture, the mass concentration of β-TCP being 8%. The preparation process included the following stages: the composite was heated on a water-bath, then it was divided into 4 equal parts, and each part was mixed separately with β-TCP for 10 min followed by joining the parts together. The total mixture density was 1.22 g/ml.

### Assembling the phantom parts

Food plastic wrap placed between the resin and the composite was used as the membrane of a replaceable inset. Filling the recess in the vertebral body was performed in the distilled water vessel. All components were embedded into water, the mixture being carefully rammed, and the model was taken out of the water (Figure 1 (b)). It enabled to prevent air bubbles from getting inside the phantom. After that the vertebra was covered by the plate and glued over with copper foil (Figure 1 (c)).

The obtained model was scanned using a computed tomography scanner Revolution EVO (General Electric, USA). The scanning parameters were the following: voltage — 80, 100, 120, 140, kV; current rate — 200 mA; the section thickness — 0.62 mm; exposure/radiation dose — 10.14 mGy. The vertebra was embedded into the water vessel to reduce the beam stiffness enhancement effect [10] and create the radiation absorption conditions close to those in the human body.

**Figure 2.**
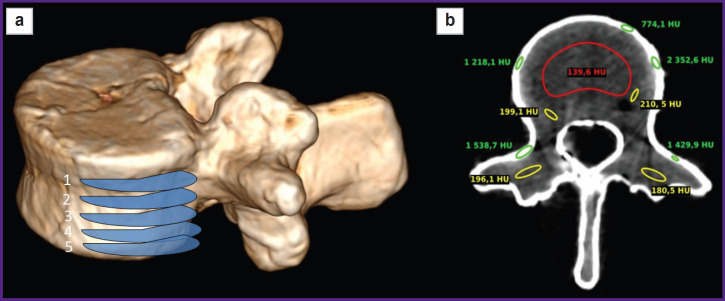
Multiplanar model reconstruction (a) and mid-axial section (at level 3 Figure (a)) with an indication of X-ray density (b) (a) numbers 1–5 indicate the sections, which underwent X-ray density measurement; (b) for visual effect, the regions of interest are marked by different colors: spongy substance — red, the vertebral base of photopolymer resin — yellow, cortical layer — green

The vertebral model underwent multiplanar reconstruction in the program RadiAnt DICOM Viewer (Medixant, Poland) (Figure 2 (a)).

The obtained CT images were analyzed using the software Weasis DICOM Viewer. There was measured mean X-ray density (XD) of three model layers according to Hounsfield intensity scale (HU) in three mutually perpendicular planes on five sections (Figure 2 (b)).

For spongy substance we compared the preset BMD with calculated values obtained considering independent asynchronous calibration curves for PHK FK2 phantom. In accordance with the data we calculated mean μ and mean square deviation (MSD) σ:

μ=1n∑i=1nхi;

σ=∑i=2n(хi−μ)2n−1,

where *х_i_* — measured values for *i* measurement, *n* — total number of measurements.

The vertebral compression deformity degree was determined in RadiAnt DICOM Viewer (Medixant, Poland) program by measuring the vertical dimensions (ventral, medial and dorsal) of the vertebral body in the sagittal plane.

The actual model dimensions were measured using a sliding caliper with depth gauge 250 mm MATRIX 316335 (Russia). After that the findings were compared with the actual measurements, and the relative error (() was determined according to the formula:

δ=х−х0х0⋅100%,

where *х*_0_ — real size of a model, *х* — dimension obtained in RadiAnt DICOM Viewer (Medixant, Poland).

A compression deformity degree according to Genant classification is characterized by index *G* calculated according to the formula [11]:

G=maxha,hm,hp−minha,hm,hpmaxha,hm,hp⋅100%,

where *h_a_* — ventral vertebral dimension, *h_m_* — medial vertebral dimension, *h_p_* — dorsal vertebral dimension.

## Results

**Figure 3.**
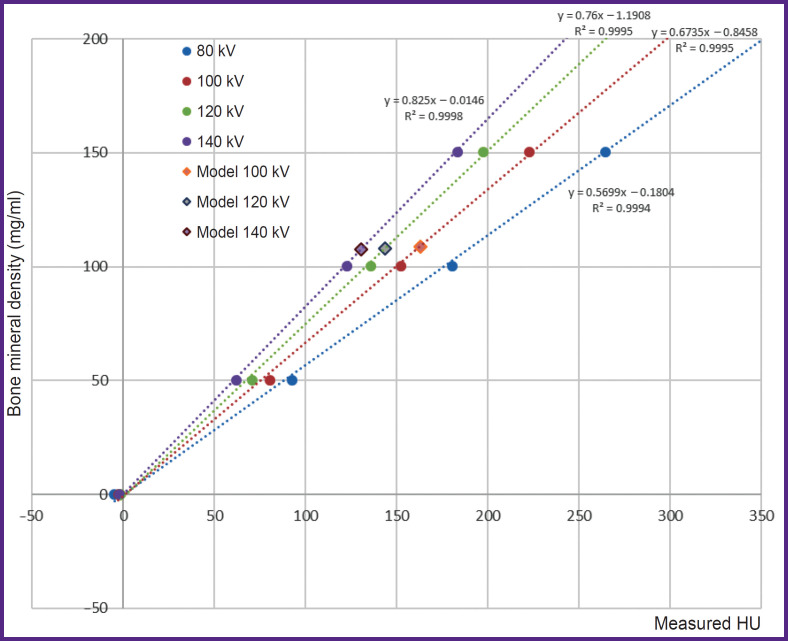
The graph of BMD dependence on measured characteristics in HU in X-ray tube voltage 100, 120 and 140 kV

Figure 3 represents the graph of BMD dependence on measured characteristics in HU; the graph was obtained in different X-ray tube voltage values.

Table 1 demonstrates XD values of all model layers and the expected XD characteristics.

**T a b l e 1:** The model measurements in voltage 120 kV

Characteristics	Spongy substance	Base	Cortical layer
Color, see Figure 2 (b)	Red	Yellow	Green
Expected X-ray density (HU)	150	200	1000
Measured X-ray density μ (HU)	143.7	196.5	1462.7
Mean square deviation σ (HU)	3.96	12.4	57.23

The averaged XD values of the spongy substance were 154.4±41.9, 162.9±18.8, 143.7±12.8, 130.6±11.0 HU (the mean value ± MSD according to the measurement data) for X-ray tube voltage 80, 100, 120 and 140 kV, respectively. Considering significant MSD value and the presence of artefacts from metal foil, for further analysis we disregarded the measurements taken in 80 kV. MSD of the measured XD values of the vertebral body, spongy substance and the cortical layer in X-ray tube voltage 120 kV appeared to be 12.40, 3.96, and 57.23 HU, respectively. The mean absolute error of determining the mineral density of the spongy substance for three X-ray tube voltages (100, 120, 140 kV) was 7.4 mg/ml, the mean relative error — 7.3% (variation coefficient). The correction coefficient to recalculate mineral density was 7 mg/ml. Considering the correction coefficient, the mean absolute error was 0.4 mg/ml, and the mean relative error — 0.4% (variation coefficient).

Table 2 represents the comparative data on the vertical dimensions of the developed model.

**T a b l e 2:** Comparison of vertical model dimensions with those obtained in CT scanning

Characteristics	Real (mm)	Measured (mm)	Relative error δ (%)
Ventral dimension	30.1	31.2	3.6
Medial dimension	29.9	30.7	2.7
Dorsal dimension	34.1	35.1	2.9
Compression deformity degree (%)	12.3	12.5	1.6

## Discussion

The present study resulted in developing L1 vertebral model with XD values comparable to those of the real human vertebra. The suggested approach can be used to produce the phantom of the entire spine or its part. The application of anthropomorphic vertebrae will enable to improve the construction of densitometric PHK FK2 phantom.

In literature there is the information on several test-objects, which contain vertebrae [12–16]. The closest analogue is Hologic DPA/QDR-1 Anthropomorphic Spine Phantom [13] designed for equipment calibration when performing dual energy X-ray densitometry. It consists of anthropomorphic models of lumbar vertebrae L1–L4, which are made of calcium hydroxyapatite modulating MBD value 1.02 g/cm^2^. Epoxide resin is used as the environment imitating soft tissues containing 60% fat. However, compared to PHK FK2, the analogue lacks the range of BMD values; therefore, it cannot be used for cross-calibration. Another disadvantage is the fact that the vertebral configuration and the environment around them are not subject to change, therefore, it is impossible to modulate different conditions of scanning and X-ray radiation absorption.

In the market there is also Hologic Anthropomorphic Spine Phantom (HolxASP(v2)), which is an upgrade version of the previous prototype [17]. The phantom has four vertebrae L1–L4 made of cast aluminium (modulating MBD value 1.009 g/cm^2^) in a plastic box. The disadvantages of the model are similar to those found in the first version of the prototype.

It should be noted that public sources give no data on the main stages of vertebral production; therefore, the present study is of great relevance. The phantom produced using the developed vertebral model will significantly differ from the existing analogues. Its advantage is in the possibility to modulate the entire MBD range of spongy substance: from osteoporosis to norm; the configuration of vertebrae and the composition of the medium surrounding them can be easily changed when required. Using the suggested phantom, it is possible to perform cross-calibration of X-ray densitometers, the training of AI systems to automatically measure XD of spongy substance and calculate the compression deformity degree of vertebral bodies [2]. Knowing the true dimension of a vertebral model, it is possible to determine the accuracy of the detecting by AI algorithms in DICOM-images.

The restrictions of the developed model application include the following:

the absence of the possibility to accurately model XD of the cortical layer due to the difficulty in choosing the material, and resulting in the large deviation of XD value of the area when scanning;
conditionality in dividing the vertebral body into the base and spongy substance, since the reconstruction was manually performed, disregarding specific anatomical structure of a particular vertebra;
marked artifacts from using metal foil.

Further, the authors are planning to improve a test-object and eliminate the specified shortcomings.

The developed technique enables to model L1, and in the future it can be adapted to develop other vertebrae: cervical, thoracic, lumbar, sacral and coccygeal.

## Conclusion

The present study suggested the technique to produce L1 vertebral model to improve the previously developed densitometric PHK FK2. CT scanning findings of the presented model confirmed the compliance of the measured XD values and the expected results. The integration of the anthropomorphic vertebra with the previous phantom version will enable to exceed the existing analogues, and can be widely used to develop new methods of densitometric diagnosis and the control of their quality.
